# Association between caregiver ability and quality of life for people with inflammatory bowel disease: The mediation effect of positive feelings of caregivers

**DOI:** 10.3389/fpsyg.2022.988150

**Published:** 2022-10-04

**Authors:** Ning Fang, Haijun Deng, Tian Fu, Zinan Zhang, Xiuyan Long, Xiaoyan Wang, Li Tian

**Affiliations:** ^1^Department of Gastroenterology, The Third Xiangya Hospital of Central South University, Changsha, Hunan, China; ^2^Department of Statistics, Guizhou University of Finance and Economics, Guiyang, Guizhou, China

**Keywords:** caregiver, IBD, quality of life, positive feeling, caring ability

## Abstract

Inflammatory bowel disease (IBD) is an incurable digestive disease. Since patients have to live with it, improving patients’ quality of life is important. Caregiver’s positive feelings and closeness may have a positive effect on patients’ quality of life. We hypothesized that caregiver’s positive feeling affected patient’s quality of life through caregiver’s caring ability, and closeness might be the upstream of this chain. In this study, we conducted a single-center cross-sectional survey by questionnaire in China to tested the hypothesis. A total of 181 patient-caregiver pairs were included. The short version of the IBD questionnaire (SIBDQ), the twelve-item short-form health survey (SF-12), the positive aspects of caregiving (PAC) and Capacity Scale of caregivers were used to collect data. All the data were collected in one interview. Spearman correlation and Bootstrap method were used to analyze the data. Mediation analysis results indicated that caregiver’s caring ability mediated the association between caregiver’s positive feelings and patients’ quality of life (*p* < 0.01), which explained 34.1% of the total variation of patients’ quality of life. Mediation analysis results also revealed that patient-evaluated or caregiver-evaluated closeness had a positive effect on patients’ quality of life through caregiver’s positive feeling and caregiver’s caring ability (*p* < 0.05), which explained 2.1 and 2.3% of the variation of patients’ quality of life. Caregiver’s positive feelings were related to caregivers’ quality of life (*p* < 0.01), but there was no significant association between caregivers’ ability and caregivers’ quality of life. In summary, our model revealed that caregiver’s positive feeling affected patients’ quality of life through caregiver’s caring ability.

## Introduction

Inflammatory bowel disease (IBD) is a chronic digestive disease, including Crohn’s disease (CD) and ulcerative colitis (UC) (Kaplan, 2015). As an incurable disease, IBD brings a negative impact on patients and their families. Lifelong drug maintenance and surgery place heavy burden on patients’ quality of life. Since patients have to live with the disease, improving patients’ quality of life has become a new focus of IBD treatment (Kaplan, 2015).

Health related quality of life (HRQoL) is a critical measurement of quality of life, which plays a major character in IBD treatment evaluation (Kaplan, 2015). It’s been proved that IBD has a negative impact on patients’ HRQoL (Liu et al., 2018). Disease activity is the main reason for declined patients’ HRQoL, since it brings clinical symptoms (Knowles et al., 2018). However, disease activity cannot explain all the variance of HRQoL. Some protective factors, such as psychotherapy, can improve patients’ HRQoL (Paulides et al., 2021). According to the bio-psycho-social model, these factors bring hope for raising HRQoL and play an important role in IBD treatment (Graffigna et al., 2021).

Caregivers’ positive feeling is one of these protective factors. Caregivers would experience a variety of positive feelings, such as satisfaction, gratification, and a bond with family. These positive feelings not only help to reduce the burden on caregivers, but also benefit patients; actually, there appears to be a virtuous circle between the positive feelings of the caregiver and the wellbeing of the patient. Previous study showed that a sense of gratification was felt when family caregivers were satisfied with their efforts in improving the wellbeing and functional status of the patients with dementia (Yu et al., 2018). The positive feelings of caregivers were associated with stronger caring ability and longer caring time, which help improve wellbeing of patients with dementia and stroke (Skolarus et al., 2017; Bayly et al., 2021). However, literatures on the relationships between caregivers’ positive feelings and IBD-related HRQoL were relatively lacking.

Closeness is often defined as the perceived psychological proximity between two people, it is a measure of the degree of dependence between two individuals and can be described in terms of including other in the self in a relationship (Berscheid et al., 1989; Aron et al., 1991; Gino and Galinsky, 2012). In terms of caregiver and patient, caregivers who were closely bonded to their patients generally reported more positive feelings, and their caregivers had better clinical outcomes (Gino and Galinsky, 2012). It suggests that the connection, or closeness, between the caregiver and the patient may have a positive impact on the patient’s HRQoL.

Caring ability, defined as the skill and ability to fulfill the need of patients, has a significant impact on patients’ quality of life. Current evidence suggests that patients with asthma can benefit from excellent caring skills (Fasola et al., 2022). Patients with IBD often suffer from symptoms such as abdominal pain and fatigue. Thus, the help from a competent caregiver can be helpful (Prince-Paul, 2021). In addition, caring ability is an external factor, but it is closely related to the quality of life of patients, and may play an important mediating role between the external environment and patients.

**FIGURE 1:**
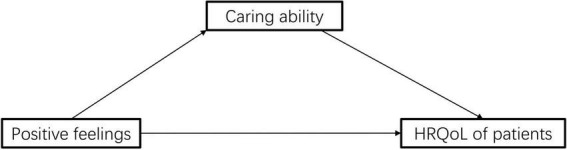
Schematic diagram of the pathway of positive feelings–caring ability–patient’s quality of life.

**FIGURE 2:**
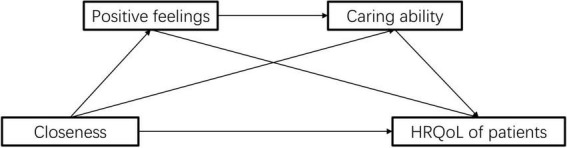
Schematic diagram of the pathway of closeness–positive feelings–caring ability–patient’s quality of life.

However, the mechanisms underlying the protective effect of caregivers’ positive feelings has not been systemically evaluated. What’s more, no study has explored the association between caregiver positive feelings and HRQoL in patients with IBD, let alone the mechanisms involved. In this study, we hypothesized that the positive feelings of caregivers could improve the HRQoL of patients with IBD by improving the caring ability of caregivers. In addition, we also tried to explore the role of closeness in this chain. In present study, we tested the hypothesis through mediation analysis. Figures 1, 2 presents the conceptual model.

## Materials and methods

### Ethical considerations

The research proposal had been approved by the Ethics Committee of the Third Xiangya Hospital of Central South University (No. I 21013). Written inform consent was obtained from each participant of this survey.

### Participants

This cross-sectional survey by questionnaire was conducted in the Third Xiangya Hospital of Central South University to explore the effect of the relationship between patients with IBD and their caregivers on patients’ HRQoL. All the eligible patients with IBD and their caregivers were randomly invited from September 2020 to December 2021. Inclusion criteria for patients with IBD were (1) voluntary participation and (2) having sufficient ability to understand the questionnaire. The inclusion criteria for caregivers were: (1) voluntary participation; (2) being a primary caregiver of patients; (3) having sufficient ability to understand the questionnaire. All the participants were taught systematically to make sure they could fully understand each item in the questionnaire.

### Instruments

#### Demographic information, clinical characteristics, and closeness assessment

Demographic information could be a confounding factor in this survey. Thus, a demographic questionnaire was made to investigate the gender, age, marital status, residence, income, and education level of patients and their caregivers. For caregivers, information such as knowledge about IBD and the caring experience was also collected. In this survey, participants’ age was divided into three levels according to the WHO age classification standard; participants’ income was divided into four levels (low, relatively low, relatively high, and high) according to Chinese subsistence allowance and individual income tax rate standard.

Disease characteristics could be confounding factors in this survey. Thus, clinical information was extracted from patients’ medical records, including the disease duration, age at diagnosis, disease location, extra intestinal manifestation, medication, co-morbidity, and surgical history. In this survey, age at diagnosis was divided into three levels according to Montreal classification; disease duration was divided into three levels according to previous studies.

The closeness between patients with IBD and their caregivers was an exposure factor in this survey. Since there was currently no suitable scale to assess it, we asked patients and caregivers to complete a scoring item respectively, in which the relationship closeness between patients and caregivers was rated on a 5-point Likert scale ranging from “totally not close (*n* = 0)” to “very close (*n* = 4).” All participants would listen to a brief instruction that explained the concept of closeness and choose the level of closeness with their caregivers/care-recipients. Our 5-point Likert scale has been shown to correlate well with the Inclusion of Other in the Self scale (Spearman correlation coefficient = 0.602, *p* < 0.01) (Aron et al., 1991); the English version of the 5-point Likert scale and further validation data is showed in Supplementary File 1.

#### Disease activity

The Crohn’s disease activity index (CDAI) score was used for patients with CD and the Mayo score was used for patients with UC (Harvey and Bradshaw, 1980; Schroeder et al., 1987). The score of each patient was given by corresponding physician in charge. CDAI scores divided patients with CD into three categories (patient with quiescent disease, active disease, and extremely severe disease) and Mayo divided patients with UC into four categories (patient with inactive disease, mild disease, moderate disease, and severe disease). To perform the following analysis, we converted Mayo scores of patients with UC, matched them with CDAI scores and classified patients with UC to three categories.

#### Quality of life questionnaire of patients with inflammatory bowel disease

The quality of life of patients with IBD was an outcome variable in this survey and measured with the short version of the IBD questionnaire (SIBDQ). SIBDQ is a self-reported scale that evaluates the quality of life of patients with IBD (Irvine et al., 1996; Jowett et al., 2001). SIBDQ consists of 10 items and 4 subscales (physical, social, emotional, and systemic). Each item is rated on a 7-point Likert scale ranging from “always” (score = 1) to “never” (score = 7). Higher scores represent better quality of life. Total scores range from 10 to 70. SIBDQ had been validated in Chinese patients with IBD (Liu et al., 2018).

#### Twelve-item short-form health survey

The quality of life of caregivers was an outcome variable in this survey and measured with the twelve-item short-form health survey (SF-12). SF-12 is a self-administered scale to measure HRQoL (Ware et al., 1996; Jenkinson et al., 1997; Gandek et al., 1998). SF-12 includes 12 items and 2 subscales (physical composite scale and mental health composite scale). Higher scores represent better quality of life. SF-12 showed excellent validity and reliability in Chinese people (Ya-Ping, 2021).

#### Positive aspects of caregiving

The positive feeling of caregivers was one mediating factor in this survey, which was measured using the Positive aspects of caregiving (PAC). PAC is a self-reported scale to measure the positive feeling of caregivers. The scale consists of 9 items and 2 dimensions (self-affirmation and life outlook) (Tarlow et al., 2004). Each item is ranged from “strongly disagree (1 point)” to “strongly agree (5 points).” The total score ranged from 9 to 45. Higher scores represent more positive feelings caregivers experienced. The Chinese version of PAC showed good reliability and validity (Zhang Rui, 2007).

#### Capacity scale of caregivers

Caring ability was one mediating factor in this survey, which was measured using the Capacity scale of caregivers. Capacity scale of caregivers is a self-reported scale that measures caring ability. It consists of 25 questions with 5 dimensions, including adaptation to the role of caregiver (5 items), response and assistance (5 items), handling with emotions (5 items), evaluation of family and community resources (5 items), and adjustment of life (5 items). Each item is ranged from “easy (0 point)” to “very difficult (2 points).” The total score ranged from 0 to 50. Higher scores represent weaker caring ability. It has been widely used in China and showed excellent reliability (Baoxiang and Xueying, 2013).

### Statistical methods

Demographic and clinical variables were presented with means (standard deviation), and categorical variables were presented with rates (percentage). *P* < 0.05 was seen as statistically significant. Missing demographic variables were filled with mode. Incomplete scales would be excluded from the statistical analysis, along with corresponding demographic information (whether completed or not).

We used Spearman correlation analysis to assess the associations among relationship, positive emotions, caring abilities, and HRQoL. Before conducting mediation analysis, control variables were filtered to eliminate their influence: Data was grouped according to baseline characteristics and compared the main variables (including relationship, positive emotion, caring ability, and HRQoL) using *t*-test, chi-square test, Wilcoxon rank-sum test, or *H*-test. If any of the main variables exhibited significant differences between groups, the grouping variable would be used as the control variable in mediation analysis. The comparison of measurement data with homogeneous variance was performed by *t*-test, and the comparison of measurement data with unequal variance was performed by the Mann–Whitney *U* test; the comparison of binary categorical variables was performed by the chi-square test, and the comparison of multivariate categorical variables was performed by the Kruskal–Wallis *H* test.

The Bootstrap method were used for mediation analysis. The Bootstrap method can estimate the proportion and significance of the mediation effect of the target pathway through repeated sampling (Hayes, 2009). The proportion of mediation effect referred to the percentage of the change of dependent variable that could be explained by mediation effect in the targeted pathway, and its significance was judged by whether the 95% confidence interval (CI) crosses zero. The Bootstrap method has become a commonly used mediation analysis method because of its high efficiency (Stine, 1990; Wen et al., 2010).

The data were analyzed using SPSS version 23.0 software (SPSS Inc., Chicago, IL, United States). Mediation analysis was performed using PROCESS for SPSS, which is available at http://www.processmacro.org/index.html.

## Results

### Participant characteristics

In our study, a total of 213 patient-caregiver pairs were randomly invited, of which 200 pairs accepted the invitation. Thirteen pairs rejected our invitation for (1) lacking time (*n* = 11) or (2) poor health condition (*n* = 2) to complete the questionnaire. Nineteen pairs were excluded from the survey for (1) incomplete questionnaire or (2) logical contradiction in the questionnaire. The effective response rate was 93%.

Demographic and clinical characteristics of participants were summarized in Table 1. The majority of caregivers were female. The mean age of caregivers was 43.7 ± 13.2 years. The majority of patients with IBD were male. The mean age of patients with IBD was 34.9 ± 11.4 years. Among the 181 patients with IBD, 53% of them were diagnosed as CD and 65% of them were in active stage.

**TABLE 1:** Demographic and clinical characteristics of patients with inflammatory bowel disease (IBD) and their caregivers.

	Patient cohort (*n* = 181)	CD cohort (*n* = 97)	UC cohort (*n* = 84)	Caregiver cohort (*n* = 181)
**Gender**				
Male, *n* (%)	102 (56)	56 (58)	46 (55)	65 (36)
**Age**				
18–44 years, *n* (%)	141 (78)	75 (77)	66 (79)	91 (50)
45–60 years, *n* (%)	35 (19)	19 (20)	16 (19)	63 (35)
Over 60 years, *n* (%)	5 (3)	3 (3)	2 (2)	24 (13)
Missing, *n* (%)	0 (0)	0 (0)	0 (0)	3 (2)
**Educational background**				
Junior high school and below, *n* (%)	12 (7)	8 (8)	4 (5)	50 (28)
Senior high school, *n* (%)	45 (25)	25 (26)	20 (24)	49 (27)
College, *n* (%)	70 (38)	34 (35)	36 (43)	46 (25)
Postgraduate and above, *n* (%)	47 (26)	25 (26)	22 (26)	20 (11)
Missing, *n* (%)	7 (4)	5 (5)	2 (2)	16 (8)
**Residence**				
City, *n* (%)	120 (66)	62 (64)	58 (69)	
Village, *n* (%)	61 (34)	35 (36)	26 (31)	
**Disease duration(year)**				
<1 year, *n* (%)	58 (32)	34 (35)	24 (29)	
1–5 years, *n* (%)	85 (47)	46 (47)	47 (56)	
≥5 years, *n* (%)	38 (21)	17 (18)	13 (15)	
Disease activity*				
Remission, *n* (%)	64 (35)	33 (34)	31 (37)	
Active, *n* (%)	87 (48)	46 (47)	41 (49)	
Severe, *n* (%)	30 (17)	18 (19)	12 (14)	
**Location**				
**UC**				
Proctitis, *n* (%)			2 (2)	
Left-sided colitis, *n* (%)			14 (17)	
Colon-sparing colitis, *n* (%)			68 (81)	
**CD**				
Ileal disease, *n* (%)		23 (24)		
Colonic disease, *n* (%)		6 (6)		
Ileocolic disease, *n* (%)		64 (66)		
Upper gastrointestinal involvement, *n* (%)		4 (4)		
**Surgical history**				
Yes, *n* (%)	21 (12)	16 (16)	5 (6)	

Values are expressed as the number of patients (%). CD, Crohn’s disease; UC, ulcerative colitis. *The Crohn’s disease activity index (CDAI) score was used for patients with CD and the Mayo score was used for patients with UC (Harvey and Bradshaw, 1980; Schroeder et al., 1987). To perform further analysis, we converted Mayo scores of patients with UC, matched them with CDAI scores and classified patients with UC to three categories.

### Preliminary correlation of main variables

Means, standard deviations, distribution characteristics, and bivariate correlations for main variables (kinship, closeness, positive feelings, caring ability, and patients’ quality of life) were shown in Table 2 and Supplementary Table 1. The association among closeness, positive feelings, caring ability, and the patients’ quality of life was found to be statistically significant (*P* < 0.01). This satisfies the preconditions for following mediation analysis.

**TABLE 2:** Correlation between multiple main variables in pathways.^+^

	Correlation coefficient^a,b^		
The main variables	Positive feelings	Caring ability	Patients’ quality of life
Closeness evaluated by patients	0.32**	−0.25**	0.49**
Closeness evaluated by caregivers	0.19**	−0.25**	0.33**
Kinship	–0.09	–0.01	–0.05
Positive feelings		−0.29**	0.35**
Caring ability			−0.48**

a: **p < 0.01. b: Since the score in the score table of caring ability is a low priority indicator, the negative value of the corresponding coefficient means positive correlation. ^+^: There were three pathways in this study: positive feeling-caring ability-patient’s health related quality of life (HRQoL) pathway, patient-evaluated closeness-positive feeling-caring ability-patient’s HRQoL pathway, and caregiver-evaluated closeness-positive feeling-caring ability-patient’s HRQoL pathway.

### Mediation effect analysis

#### Positive feeling-caring ability-patient health related quality of life pathway

Since positive feelings, caring ability, and patients’ HRQoL showed significant correlations (Table 2), the Bootstrap method was used to examine whether caring ability mediates the association between positive feelings and patients’ HRQoL. We took caregivers’ gender, residence, and disease activity as control variables, caregivers’ positive feelings as independent variables, caring ability as mediating variables, and patients’ HRQoL as dependent variables to perform mediation analysis. The results showed that the mediation effect of caring ability between positive feelings and patients’ HRQoL was significant (95% CI: 0.09–0.44, Table 3). We also confirmed these results with the Sobel’s test (Supplementary File 2).

**TABLE 3:** Bootstrap method evaluated mediation effect and significance of three pathways.

Pathways	Specific indirect effect	95% CI	Specific/Total effect
Positive feelings–caring ability–patient’s HRQoL	0.24 ± 0.09	0.09, 0.44	34.1%
Caregiver evaluated closeness–positive feelings–caring ability–patient’s HRQoL	0.11 ± 0.06	0.02, 0.29	2.3%
Patient evaluated closeness–positive feelings–caring ability–patient’s HRQoL	0.10 ± 0.06	0.01, 0.26	2.1%

Specific indirect effect: Change of dependent variable that can be explained by the mediation effect in the pathway. 95% CI: If the 95% CI does not include 0, the mediation effect is statistically significant. Specific/Total effect: The proportion of mediation effect of targeted pathway to the total variation of patients’ quality of life.

#### Closeness-positive feeling-caring ability-patient health related quality of life pathway

Since patient-evaluated closeness, positive feelings, caring ability, and patients’ HRQoL showed significant correlations, the Bootstrap method was used to test whether positive feelings and caring ability mediate the association between closeness and patients’ HRQoL. We took caregivers’ gender, residence, and disease activity as control variables, caregivers’ positive feelings as independent variables, caring ability as mediating variables, and patients’ HRQoL as dependent variables to perform mediation analysis. The results showed that the mediation effect in the pathway of patient-evaluated closeness-positive feelings-caring ability-patients’ HRQoL was significant (95% CI: 0.02–0.29, Table 3). The mediation effect in the pathway of caregiver-evaluated closeness- positive feelings-caring ability-patients’ HRQoL was significant (95%CI: 0.01–0.26, Table 3). We also confirmed these results with the Sobel’s test (Supplementary File 2).

### Caregivers’ positive feelings, caring ability, and quality of life

Physical composite subscale (PCS-12) and mental health composite subscale (MCS-12) of SF-12 reflected the physical and mental health of the subjects, respectively. Mean PCS-12 score and mean MCS-12 score of caregivers were 37.0 ± 12.3 and 20.0 ± 15.3, respectively. Correlation analysis showed that positive feelings were related to PCS-12 (*r* = 0.27, *p* < 0.01) and MCS-12 (*r* = 0.21, *p* < 0.01), but there was no significant association between caring ability and PCS-12 (*p* = 0.08) or MCS-12 (*p* = 0.28). Patient’s HRQoL was related to PCS-12 (*r* = 0.16, *p* = 0.03), but showed no significant association with MCS-12 (*p* = 0.07).

## Discussion

Our study firstly explored the relationship between caregivers’ positive feelings and patients’ HRQoL in IBD, and took caregivers’ caring ability as mediation factors to explain the correlation between caregivers’ positive feelings and patients’ HRQoL. We also explored the role of closeness in this chain.

As expected, we found that caregivers’ positive feelings were positively correlated with patients’ HRQoL in IBD. Specifically, the more positive feelings the caregiver experienced, the higher the patients’ HRQoL was. This clarifies the possibility that positive feelings are not only protective factors for caregivers’ psychological status, but also for patients’ HRQoL (Yu et al., 2018).

The mediation analysis supported the idea that positive feeling had a positive effect on HRQoL through caring ability. Our study showed that positive feeling was positively associated with caring ability, which was in line with of previous study (Cheng et al., 2019; Bayly et al., 2021; Li-li et al., 2021). Caregivers with a higher level of positive feelings were better at realizing their personal values. Therefore, they tended to learn disease-related knowledge and skills through multiple channels, which promoted the improvement of caring ability (Xue et al., 2018). We speculated that positive feelings might bring strength and push caregivers to raise their caring ability. At present, no study has directly shown a link between positive feelings of caregivers and their caring abilities in IBD, but family caregivers of patients with IBD in previous studies have shown an urgent desire to understand the disease (Lorente and Jimenez, 2018; Carlsen et al., 2019; Garcia-Sanjuan et al., 2019). What’s more, our results showed a significant association between caring ability and patients’ HRQoL. Since caring ability is defined as the skill and ability to fulfill the need of patients, this association is natural (Clark and Rakowski, 1983). Excellent caring skills are important for patients with IBD: Patients with IBD in active period suffer from abdominal pain and diarrhea, and often experience fatigue and anxiety. Skilled caregivers understand these symptoms, and can provide targeted help and emotional support to help patients with IBD overcome obstacles (Fragoso and Rodrigues, 2020; Vigano et al., 2021).

What’s more, the mediation analysis supported the idea that closeness had a positive effect on HRQoL through positive feelings and caring ability. Our result showed that a better relationship between patients with IBD and caregivers might bring more positive feelings to caregivers. This is in line with previous studies, which show that caregivers would experience satisfaction, resilience, and self-growing in caring for their spouse (Wang et al., 2017; Aloweni et al., 2019). Although these studies did not assess closeness systematically, participants reported excellent relationship quality, which indicated that closeness was the core of bringing positive feelings. Although the mediation effect of the closeness- positive feelings–caring ability–patient’s quality of life pathway was significant, it could only explain approximately 2% of the total variation of patient’s HRQoL. Possible reason is that there are two mediators in the chain mediation analysis, and the strength of the mediating effect is weakened during the transmission process. We will add moderators to the model in future studies to optimize it.

We also found that positive feelings were associated with caregivers’ HRQoL, but there was no significant association between caregiving ability and caregivers’ HRQoL. This is consistent with previous results (Wittenberg et al., 2021). Thus, it could be valuable in introducing positive emotion unraveling into the psychological support training courses for caregivers of patients with IBD (Shukla et al., 2018).

When interpreting the results of this study, its limitations should be taken into account. First of all, this study was a single-center study; the participants were mainly Asians from 18 to 60, mainly active period patients, which might limit the application of the results. In addition, this study was a cross-sectional study. Thus, causality cannot be presumed through mediation analysis. Our study could only provide a basis for the possible causal chain. Longitudinal studies are still needed to determine whether the chain exists. In addition, the caring ability could only explain part of the variation caused by positive feelings; there were still other factors to be explored. Finally, closeness was measured using a 5-point Likert scale in our study, for there was currently no accepted scale for measuring closeness between caregivers and patients. Therefore, it is necessary to fully consider its limitations when interpreting results related to closeness.

In conclusion, this study proposed a model to explain the important role of positive feelings in IBD treatment. The model showed that positive feelings have an impact on HRQoL through caring ability. We believe that the positive feelings caregivers gain from their caregiving work can encourage them to improve their caregiving skills and improve HRQoL of patients with IBD. Therefore, we recommend including positive feelings unraveling in caregiver education of IBD.

## Data availability statement

All the raw data supporting the conclusions of this article is available from the corresponding authors.

## Ethics statement

The studies involving human participants were reviewed and approved by the Ethics Committee of the Third Xiangya Hospital of Central South University. The patients/participants provided their written informed consent to participate in this study.

## Author contributions

NF analyzed data and wrote the manuscript. HJD designed the study and helped with data analysis. TF took part in data collection and data analysis. ZNZ helped with data collection and prepared figures. XYL did the work of literature search. XYW supervised the study. LT provided critical revision of the manuscript for important intellectual content. All authors contributed to the article and approved the submitted version.
