# Supplementary material for: Association between caregiver ability and quality of life for people with inflammatory bowel disease: The mediation effect of positive feelings of caregivers
Source: Front Psychol. 2022 Oct 4;13:988150. doi: 10.3389/fpsyg.2022.988150 (PMC9577491; doi:10.3389/fpsyg.2022.988150)
Supplement: Supplementary file 1 [file Presentation_1.pdf]

Supplementary file 1:

Closeness scale

Thank you for receiving our interview. This scale is used to collect information about closeness. Closeness is often defined as the perceived psychological proximity between two people, or the degree of dependence between two individuals. It is a measure of how connected you are with others, such as with your friends, family, or loved ones. Please assess how close you are to your caregiver/care-recipient.

I would assess my close with my caregiver/care-recipient as:

1. Very not close
2. Less close
3. Generally close
4. Closer
5. Very close

Footnote: Validation test information

Aron et al. developed the Inclusion of Other in the Self scale (IOS) to measure closeness, which has good reliability and validity, and uses seven pairs of circles to represent different degrees of closeness [1-2]. However, the scale has not been validated in Chinese people, so we did not use it for investigation. To prove our scale is reliable, we performed a correlation analysis using IOS scores and our closeness scale scores. The caregiver-evaluated closeness and caregiver's IOS showed significant correlation (Spearman correlation coefficient=0.602, $p<0.01$); the patient-evaluated closeness and patient's IOS showed significant correlation (Spearman correlation coefficient=0.676, $p<0.01$).

[1]Aron, ArthurAron, Tudor E N , et al. Close relationships as including other in the self.[J]. Journal of Personality and Social Psychology, 1991.
[2]Aron A, Aron E N, Smollan D. Inclusion of Other in the Self Scale and the Structure of Interpersonal Closeness[J]. Journal of Personality & Social Psychology, 1992
