# Supplementary material for: Association between caregiver ability and quality of life for people with inflammatory bowel disease: The mediation effect of positive feelings of caregivers
Source: Front Psychol. 2022 Oct 4;13:988150. doi: 10.3389/fpsyg.2022.988150 (PMC9577491; doi:10.3389/fpsyg.2022.988150)
Supplement: Supplementary file 2 [file Presentation_2.pdf]

Supplementary file 2: the Results of the Sobel test

1. Introduction:

To ensure the stability of our results in 3.3 mediation analysis, we used the Sobel's test to analyses the mediation effect. The Sobel's test proposed by Baron and Sobel et al. is a classic method for evaluating mediation effects [1,2]. The Sobel's test first constructed a series of regression equations containing one or more variable to be evaluated. By evaluating the statistical significance of the standardized regression coefficients in each regression equation, the Sobel's test could determine whether the mediation effect of the target path was statistically significant.

2. Positive feeling-caring ability-patient HRQoL pathway

the Sobel's test was used to examine whether caring ability mediates the association between positive feelings and patients' HRQoL. Caregiver gender, residence and disease activity were used as control variables (Supplementary Table 2). Firstly, positive feelings were significantly correlated with patients' HRQoL (Regression equation 1, $\beta=0.27$, $p<0.01$). In the second step, positive feelings were significantly correlated with the caring ability (Regression equation 2, $\beta=-0.25$, $p<0.01$). In the third step, positive feelings and caring ability were significantly correlated with patients' HRQoL (Regression equation 3, $\beta=0.19$, $p<0.01$). This indicated that caring ability mediated the association between positive feelings and patients' HRQoL, with a significant mediation effect (Supplementary Table 4).

3. Closeness-positive feeling-caring ability-patient HRQoL pathway

Caregiver gender, residence and disease activity were used as control variables. Firstly, patient-evaluated closeness was significantly associated with patients' HRQoL (Regression equation 4, $\beta=0.49$, $p<0.01$). In the second step, patient-evaluated closeness was significantly associated with positive feelings (Regression equation 5, $\beta=0.30$, $p<0.01$). In the third step, patient-evaluated closeness and positive feelings were significantly related to caring ability (Regression equation 6, $\beta=0.59$, $p<0.01$). In the final step, patient-evaluated closeness, positive feelings and caring abilities were significantly associated with patients' HRQoL (Regression equation 7, $\beta=0.41$, $p<0.01$). This indicated that positive feelings and caring ability significantly mediated the relationship between patient-evaluated closeness and patients' HRQoL. We also constructed similar models for caregiver-evaluated closeness -positive feeling-caring ability-patient HRQoL pathway (Regression equation 8-11), which indicated that positive feelings and caring ability significantly mediated the relationship between caregiver-evaluated closeness and patients' HRQoL($p<0.05$, Supplementary Table 4).

References:

[1] Baron RM, Kenny D A. The moderator-mediator variable distinction in social psychological research: conceptual, strategic, and statistical considerations[J]. J Pers Soc Psychol,1986,51(6):1173-1182.

[2] Sobel M. Asymptotic Confidence Intervals for Indirect Effects in Strucutural Equation Models[J]. Sociological Methodology 1982,1982.
