# Supplementary material for: Association between caregiver ability and quality of life for people with inflammatory bowel disease: The mediation effect of positive feelings of caregivers
Source: Front Psychol. 2022 Oct 4;13:988150. doi: 10.3389/fpsyg.2022.988150 (PMC9577491; doi:10.3389/fpsyg.2022.988150)
Supplement: Supplementary file 3 [file Data_Sheet_1.docx]

| **Supplementary table 1.** Descriptive characteristics of the main indicators | | | |
| --- | --- | --- | --- |
| **Main indicators** | | **Mean±SD or frequency (%)** |  |
|  |  |  |  |
| **Patients’ quality of life** | | 44.1±13.0 |  |
| **Positive feelings** | | 23.4±6.5 |  |
| **Caring ability** | | 12.8±10.1 |  |
| **Closeness evaluated by patients** | |  |  |
| Very not close | | 11 (6) |  |
| Less close | | 12 (7) |  |
| Generally close | | 32 (18) |  |
| Closer | | 46 (25) |  |
| Very close | | 80 (44) |  |
| **Closeness evaluated by caregivers** | |  |  |
| Very not close | | 7 (4) |  |
| Less close | | 6 (3) |  |
| Generally close | | 32 (18) |  |
| Closer | | 50 (28) |  |
| Very close | | 86 (47) |  |
| **Kinship** |  |  |  |
| Parents | | 66 (36) |  |
| Spouse | | 89 (49) |  |
| Others | | 26 (14) |  |

| **Supplementary Table 2.** Comparison of mean values of mediating factors | | | | | | |
| --- | --- | --- | --- | --- | --- | --- |
| **Possible mediating factors** | **Statistics** | | | | |  |
|  | **Closeness evaluated by patients** | **Closeness evaluated by caregivers** | **Positive feelings** | **Caring ability** | **Patients’ HRQoL** |  |
| Gender (patients) | 0.27 | 0.07 | 0.78 | 0.21 | 0.56 |  |
| Gender (caregivers) | 0.35 | 0.1 | 0.68 | **0.01*** | 0.36 |  |
| Age (patients) | 0.72 | 0.34 | 0.87 | 0.83 | 0.34 |  |
| Age (caregivers) | 0.79 | 0.6 | 0.49 | 0.78 | 0.38 |  |
| Educational levels (patients) | 0.11 | 0.06 | 0.36 | 0.33 | 0.55 |  |
| Educational levels (caregivers) | 0.64 | 0.32 | 0.1 | 0.14 | 0.15 |  |
| Marital status | 0.64 | 0.37 | 0.41 | 0.79 | 0.37 |  |
| Residence | 0.41 | 0.06 | 0.64 | **0.04*** | **0.03*** |  |
| Income | 0.17 | 0.36 | 0.87 | 0.96 | 0.7 |  |
| Surgical history | 0.42 | 0.66 | 0.74 | 0.26 | 0.28 |  |
| Co-morbidity | 0.88 | 0.52 | 0.16 | 0.2 | 0.43 |  |
| Extra intestinal manifestation | 0.06 | 0.57 | 0.73 | 0.47 | 0.31 |  |
| Disease duration | 0.19 | 0.6 | 0.43 | 0.19 | 0.62 |  |
| Age at diagnosis | 0.25 | 0.43 | 0.12 | 0.78 | 0.6 |  |
| **Possible mediating factors** | **Statistics** | | | | |  |
|  | **Closeness evaluated by patients** | **Closeness evaluated by caregivers** | **Positive feelings** | **Caring ability** | **Patients’ HRQoL** |  |
| Disease subtypes | 0.74 | 0.18 | 0.69 | 0.57 | 0.63 |  |
| Disease activity | 0.06 | 0.51 | **0.01*** | **<0.01**** | **<0.01**** |  |
| The comparison of measurement data with homogeneous variance was performed by t-test, and the comparison of measurement data with unequal variance was performed by the Mann-Whitney U test; the comparison of binary categorical variables was performed by the chi-square test, and the comparison of multivariate categorical variables was performed by the Kruskal-Wallis H test. | | | | | |  |
|  | | | | | | |

**Supplementary Table 3.** Demographic and clinical characteristics of patients with IBD

|  | **Patient cohort** | **CD cohort (n=97)** | **UC cohort (n=84)** |
| --- | --- | --- | --- |
|  | **(n=181)** |  |  |
| **Income (CNY)** | |  |  |
| >2000, n (%) | 18 (10) | 10 (10) | 8 (10) |
| 2000-4999, n (%) | 75 (41) | 43 (45) | 32 (38) |
| 5000-9999, n (%) | 62 (34) | 27 (28) | 35 (42) |
| >10000, n (%) | 14 (8) | 7 (7) | 7 (8) |
| Missing, n (%) | 12 (7) | 10 (10) | 2 (2) |
| **Marital status** | |  |  |
| Unmarried, n (%) | 61 (34) | 64 (66) | 56 (67) |
| Married, n (%) | 115 (64) | 30 (31) | 26 (31) |
| Divorce, n (%) | 0 (0) | 0 (0) | 0 (0) |
| Missing, n (%) | 5 (2) | 3 (3) | 2 (2) |
| **Medication** | |  |  |
| Biologicals, n (%) | 112 (62) | 64 (66) | 48 (57) |
| 5-ASA, n (%) | 83 (46) | 56 (58) | 27 (32) |
| AZA, n (%) | 74 (41) | 41 (42) | 33 (39) |
| Glucocorticoid, n (%) | 87 (48) | 52 (54) | 35 (42) |
| **Co-morbidities** | |  |  |
| Circulation system co-morbidity, n (%) | 7 (4) | 5 (5) | 2 (2) |
| Respiratory system co-morbidity, n (%) | 3 (2) | 1 (1) | 2 (2) |
| Without co-morbidity, n (%) | 171 (94) | 91 (94) | 80 (96) |
| **Extra intestinal manifestation** | | |  |
| Oral aphthous ulcers, n (%) | 16 (8) | 8 (8) | 8 (10) |
| Ankylosing spondylitis , n (%) | 1 (1) | 1 (1) | 0 (0) |
| Arthralgia/Arthritis, n (%) | 4 (2) | 3 (3) | 1 (1) |
| Erythema Nodosum, n (%) | 3 (2) | 3 (3) | 0 (0) |
| Without extra intestinal menifestation, n (%) | 157 (87) | 82 (85) | 75 (89) |
| **Age at diagnosis** | |  |  |
| ≤16y, n (%) | 6 (3) | 4 (4) | 2 (2) |
| 17-40y, n (%) | 133 (74) | 71 (73) | 62 (74) |
| >40y, n (%) | 42 (23) | 22 (23) | 20 (24) |

Values are expressed as the number of patients (%).

CD：Crohn’s disease; UC：ulcerative colitis; CNY: Chinese Yuan; 5-ASA: 5-aminosalicylates; AZA: azathioprine

**Supplementary Table 4.** Sobel’s test evaluated mediation effect significance of three pathways^+^

| **Regression equation** | **Dependent variable** | **Independent variable** | **β** | **p** |
| --- | --- | --- | --- | --- |
| Regression equation 1 | Patient’s HRQoL | Positive feelings | 0.27 | <0.01 |
| Regression equation 2 | Caring ability | Positive feelings | -0.25 | <0.01 |
| Regression equation 3 | Patient’s HRQoL | Positive feelings | 0.19 | <0.01 |
|  |  | Caring ability | -0.28 | <0.01 |
| Regression equation 4 | Patient’s HRQoL | Patient-evaluated closeness | 0.49 | <0.01 |
| Regression equation 5 | Positive feelings | Patient-evaluated closeness | 0.29 | <0.01 |
| Regression equation 6 | Caring ability | Patient-evaluated closeness | 0.59 | <0.01 |
|  |  | Positive feelings | 0.11 | <0.01 |
| Regression equation 7 | Patient’s HRQoL | Patient-evaluated closeness | 0.41 | <0.01 |
|  |  | Positive feelings | 0.11 | 0.04 |
|  |  | Caring ability | -0.18 | <0.01 |
| Regression equation 8 | Patient’s HRQoL | Caregiver-evaluated closeness | 0.36 | <0.01 |
| Regression equation 9 | Positive feelings | Caregiver-evaluated closeness | 0.23 | <0.01 |
| Regression equation 10 | Caring ability | Caregiver-evaluated closeness | -0.27 | <0.01 |
|  |  | Positive feelings | -0.19 | <0.01 |
| Regression equation 11 | Patient’s HRQoL | Caregiver-evaluated closeness | 0.26 | <0.01 |
|  |  | Positive feelings | 0.16 | <0.01 |
|  |  | Caring ability | -0.19 | <0.01 |

+: The Sobel’s test first constructed a series of regression equations containing one or more variable to be evaluated. By evaluating the statistical significance of the standardized regression coefficients in each regression equation, the Sobel’s test could determine whether the mediation effect of the target path was statistically significant.

Regression equation 1-3 are used to evaluate positive feeling-caring ability-patient’s HRQoL pathway, Regression equation 4-11 are used to evaluate two closeness-positive feeling-caring ability-patient’s HRQoL pathways.

β: Standardized regression coefficient.
